# Quantitative analysis of the effect of radiation on mitochondria structure using coherent diffraction imaging with a clustering algorithm

**DOI:** 10.1107/S2052252521012963

**Published:** 2022-01-21

**Authors:** Dan Pan, Jiadong Fan, Zhenzhen Nie, Zhibin Sun, Jianhua Zhang, Yajun Tong, Bo He, Changyong Song, Yoshiki Kohmura, Makina Yabashi, Tetsuya Ishikawa, Yuequan Shen, Huaidong Jiang

**Affiliations:** a School of Physical Science and Technology and Center for Transformative Science, ShanghaiTech University, 393 Middle Huaxia Road, Shanghai 201210, People’s Republic of China; b State Key Laboratory of Medicinal Chemical Biology and College of Life Sciences, Nankai University, 94 Weijin Road, Tianjin 300071, People’s Republic of China; c Photon Science Division, Paul Scherrer Institute, 5232 Villigen, Switzerland; d Department of Physics, Pohang University of Science and Technology, Pohang 37673, South Korea; e SPring-8 Center, RIKEN, 1-1-1, Kouto, Sayo, Hyogo 679-5148, Japan

**Keywords:** coherent diffraction imaging, radiation damage, mitochondria, clustering algorithms, X-ray imaging, quantitative analysis

## Abstract

Radiation damage and a low signal-to-noise ratio are the primary factors that limit spatial resolution in coherent diffraction imaging (CDI) of biomaterials using X-ray sources. Introduced here is a clustering algorithm named *ConvRe* based on deep learning, and it is applied to obtain accurate and consistent image reconstruction from noisy diffraction patterns of weakly scattering biomaterials. To investigate the impact of X-ray radiation on soft biomaterials, CDI experiments were performed on mitochondria from human embryonic kidney cells using synchrotron radiation. Benefiting from the new algorithm, structural changes in the mitochondria induced by X-ray radiation damage were quantitatively characterized and analysed at the nanoscale with different radiation doses. This work also provides a promising approach for improving the imaging quality of biomaterials with XFEL-based plane-wave CDI.

## Abstract 2

A clustering algorithm based on deep learning is proposed to perform accurate image reconstruction from noisy coherent diffraction patterns. Structural changes in mitochondria induced by X-ray radiation damage are quantitatively characterized and analysed at the nanoscale with different radiation doses.

## 1. Introduction

X-ray microscopy has been used to image biomaterials for many decades due to its high penetration depth and potentially high spatial resolution. However, X-ray radiation damage is the main limitation to high-resolution imaging, and this damage depends on the X-ray energy, irradiation time, type of material, data collection temperature *etc.* (Kmetko *et al.*, 2006 ▸; Gianoncelli *et al.*, 2015 ▸). To date, several methods have been used to investigate radiation damage in X-ray imaging, such as near-edge X-ray absorption fine structure spectroscopy (Zhang *et al.*, 1995 ▸; Coffey *et al.*, 2002 ▸), X-ray fluorescence microscopy (Kosior *et al.*, 2012 ▸), scanning transmission X-ray microscopy (Williams *et al.*, 1993 ▸) and X-ray photoemission electron microscopy (Wang *et al.*, 2009 ▸). However, a lack of effective technologies that can quantitatively image the changes in biomaterials at the nanoscale during irradiation has prevented a comprehensive understanding of the effects of radiation at such length scales.

Coherent diffraction imaging (CDI) is a lensless method currently under rapid development to achieve high spatial resolution (Sayre, 1980 ▸; Miao *et al.*, 1999 ▸). By reconstructing the lost phase of the diffracted wavefield, the electron densities of samples can be characterized with high contrast (Miao *et al.*, 2003 ▸). To achieve precise reconstructions, diffraction patterns with high signal-to-noise ratio are usually necessary for CDI.

A clustering algorithm named *ConvRe*, based on deep learning, is introduced here and used to perform accurate image reconstruction from noisy coherent diffraction patterns.

Mitochondria from human embryonic kidney (HEK293) cells were chosen to study the effect of radiation on the structure of biomaterials. Mitochondria play an important role, not only in the supply of cellular energy but also in metabolic processes and cell death (Wang *et al.*, 2019 ▸). Structural changes in the mitochondrion induced by X-ray radiation damage were quantitatively characterized from the images reconstructed using our approach.

## 2. Materials and methods

### 2.1. Plane-wave mitochondria CDI experiment

**Figure 1:**
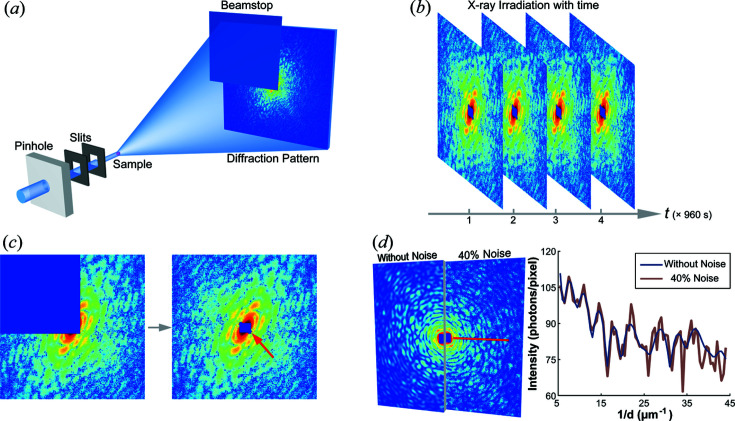
(*a*) A schematic layout of plane-wave CDI. (*b*) The four diffraction patterns from a mitochondrion after different total accumulated exposure times. (*c*) Each experimental diffraction pattern was partially blocked by a beamstop, as shown on the left. The red arrow points to the missing data remaining after enforcing centrosymmetry. (*d*) Simulated diffraction patterns with and without Poisson noise. The line scan (taken along the red line in the pattern) shows the variation in diffraction intensity.

The mitochondria used for this experiment were extracted from human embryonic kidney (HEK293) cells and chemically fixed with formaldehyde and glutaraldehyde using the procedure described by Fan *et al.* (2015 ▸). The CDI experiment was carried out on the BL29XU beamline at SPring-8 Japan. As shown in Fig. 1 ▸(*a*), during the experiment, a 10 µm diameter pinhole was placed in the incident beam, 0.35 m upstream of the sample, to ensure the necessary spatial coherence at a photon energy of 5.5 keV. Two silicon apertures were mounted downstream of the pinhole to clean stray scattering from the pinhole. The sample was placed in a chamber filled with helium and which was at room temperature. The diffraction signals were collected with a CCD detector located 1.496 m downstream of the sample position. The CCD was composed of 1340 × 1300 pixels with a single pixel size of 20 × 20 µm. To protect the CCD from the directly transmitted beam, a beamstop was placed in front of the CCD.

To enhance the dynamic range of the diffraction measurement, the diffraction intensities in the region of the pattern at low scattering angle (referred to as the low spatial frequency region of interest, LROI) were recorded separately to those at high scattering angles (the high spatial frequency region of interest, HROI) (Chapman, Barty, Marchesini *et al.*, 2006 ▸). In this experiment, the acquisition time of the LROI was 0.08 s per exposure with 1000 exposures, that of the HROI was 12 s per exposure with 80 exposures, and the total exposure time was 1040 s. To investigate the effects of irradiation on the structure of the mitochondria, another three measurements of the same sample were subsequently conducted using the same sets of exposures. In this way, four diffraction patterns were obtained with equal exposure times (and hence similar signals) but with increasing cumulative doses, as shown in Fig. 1 ▸(*b*).

As the signal-to-noise ratios of all four diffraction patterns were low, we binned 3×3 pixels into 1 pixel to enhance the signal-to-noise ratio. A deconvolution was then performed on the patterns to correct for the reduction in contrast due to the binning (Song *et al.*, 2007 ▸). The final size of each pattern was 411×411 pixels and the corresponding cumulative radiation doses to the sample were calculated, based on the experimental data, to be 30.1, 57.8, 85.6 and 113 MGy, respectively, for the four patterns (see the supporting information). The data were submitted to the Coherent X-ray Imaging Data Bank (https://www.cxidb.org; Maia, 2012 ▸).

### 2.2. Phase retrieval

**Figure 2:**
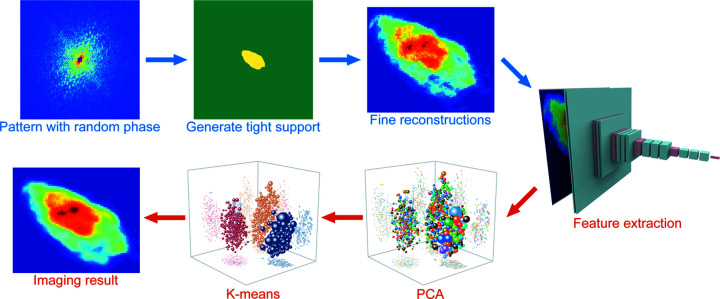
A flowchart of the whole reconstruction. Stages labelled with blue and orange text represent phase retrieval and clustering, respectively.

As shown in Fig. 2 ▸, the whole reconstruction process of the diffraction pattern is divided into two main stages, phase retrieval (blue labels) and clustering (orange labels). In this experiment, the phase information was retrieved from the diffraction pattern by the oversampling smoothness (*OSS*) algorithm designed by Rodriguez *et al.* (2013 ▸). The *OSS* algorithm, which is suitable for noisy patterns, applies different Gaussian kernels to the diffraction-space image arrays during reconstruction. After the *OSS* iterations, the four real-space reconstructions were clustered according to their structures by *ConvRe* (see details in the supporting information).

**Figure 3:**
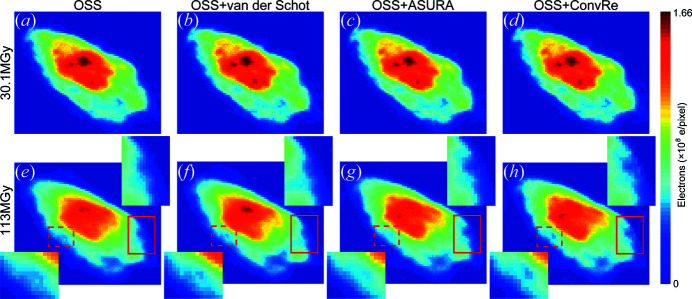
(*a*)–(*d*) Images reconstructed by different methods at a dose of 30.1 MGy. (*e*)–(*h*) Images reconstructed by different methods at a dose of 113 MGy. The images in the upper right corner are magnified views of the regions indicated by red solid rectangles, and the images in the lower left corner are magnified views of the regions indicated by the red dashed rectangles.

To evaluate the performance of *ConvRe* for noisy patterns, the methods proposed by van der Schot *et al.* (2015 ▸) and Sekiguchi *et al.* (2016 ▸) were also performed to cluster independent reconstructions, and a total of four imaging results were obtained, as shown in Fig. 3 ▸. To compare the results to assess which method is most suitable, it is not sufficient simply to compare the reconstructed images by eye, but a quantitative characterization is crucial, as discussed further below.

The resolution of images reconstructed from their coherent diffraction patterns is often characterized using the phase retrieval transfer function (PRTF), defined as the ratio of the averaged reconstructed Fourier amplitude to the experimental Fourier amplitude (Chapman, Barty, Marchesini *et al.*, 2006 ▸), according to

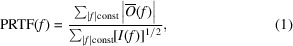

where 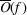 is the Fourier transform of the average of independent reconstructions (that is, iterates taken sufficiently far from each other) after first adjusting the average phase of the object to a common value, and *I*(*f*) are the measured diffraction intensities. The frequency *f* is inversely proportional to the full period resolution *d*. Here, 24 independent reconstructions were averaged and the expected lower bound was 0.2 (Ayyer *et al.*, 2019 ▸).

**Figure 4:**
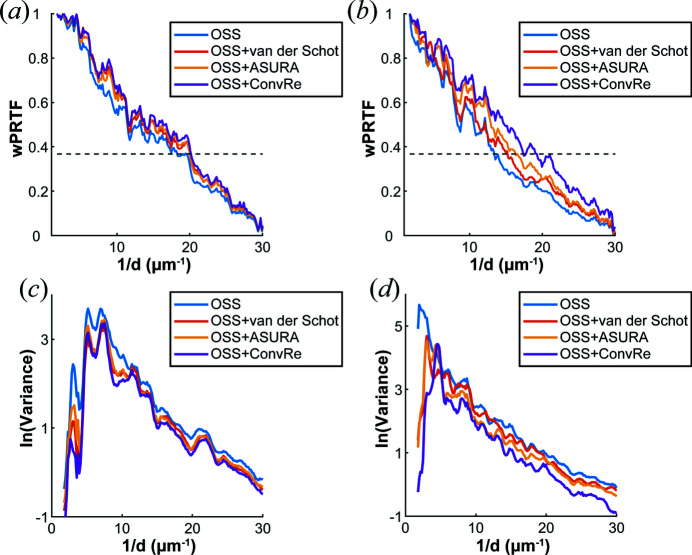
(*a*) and (*b*) The wPRTF curves of different algorithms at doses of (*a*) 30.1 MGy and (*b*) 113 MGy as a function of the resolution 1/*d*. The value of the dashed line is 1/e, which serves as the criterion for the highest achieved resolution. (*c*) and (*d*) The natural logarithmic variance of different algorithms at (*c*) 30.1 MGy and (*d*) 113 MGy.

Due to the high noise in the diffraction patterns, direct calculation of the PRTF would cause the values to increase abnormally in the high-frequency region, so the PRTF was modified by modulating it with a Wiener filter to provide an improved measure of the quality of the reconstruction (Steinbrener *et al.*, 2010 ▸),

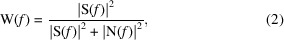

where S(*f*) is the pure scattering signal and N(*f*) is an estimation of the noise. The noise term is set to a constant where the power spectral density *I*(*f*) tends to fluctuate steadily at high frequency. As the frequency *f* increases, the proportion of noise increases, making W(*f*) lower. When the PRTF is multiplied by the Wiener filter, it is reduced by the weighting to give a better estimate of the quality of the reconstruction as a function of spatial frequency (see the supporting information). Figs. 4 ▸(*a*) and 4 ▸(*b*) illustrate the Wiener-weighted PRTF (wPRTF) curves under doses of 30.1 and 113 MGy, respectively. At the lower radiation dose, the resolution estimates obtained by all four methods were relatively high and quite close together. *OSS*+*ConvRe* reached the highest resolution of 49.7 nm, and *OSS* had the lowest resolution of 51.0 nm. However, when the radiation dose reached 113 MGy, the performance of the algorithms differed. The resolutions of *OSS*, *OSS*+van der Schot, *OSS*+ASURA (Sekiguchi *et al.* (2016 ▸) and *OSS*+*ConvRe* were 74.9, 66.1, 61.7 and 57.9 nm, respectively, showing that the clustering algorithm based on deep learning produced images of higher resolution.

In addition, the variances in the amplitudes of the diffraction patterns calculated from the 24 best reconstructed images as a function of spatial frequency were analysed for the different algorithms, as shown in Figs. 4 ▸(*c*) and 4 ▸(*d*). Fig. 4 ▸(*c*) illustrates that the variances of the three clustering methods are similar, and they are all lower than for *OSS* at a radiation dose of 30.1 MGy. For all methods, the variance is seen to be small at frequencies less than 3 µm^−1^, which means that there is almost no difference in the shape of the reconstructed images. As the frequency increases from this level, the variance values first increase, and differences could also be observed in real space. As the frequency increases further, the variance values decrease again. This is because there is a difference in magnitude between high-frequency and low-frequency signals. Even if the difference increases, the values of variance in high-frequency regions will not be larger than those in low-frequency regions. Plots of the variance as a function of frequency at 113 MGy are shown in Fig. 4 ▸(*d*), and significant differences are apparent in the variance plots between the different algorithms. The *OSS* algorithm exhibits a larger variance at extremely low frequencies, indicating that the simple *OSS* algorithm, without any clustering, is not suitable for reconstructing patterns with such a low signal-to-noise ratio. After using the clustering algorithms, the variances are significantly reduced at frequencies less than 5 µm^−1^, indicating that all clustering algorithms could distinguish the shape of the object well. Similar to the case of the lower radiation dose, the variances are seen to increase as the frequency increases. At frequencies above 10 µm^−1^, the variance of *OSS*+*ConvRe* is found to be the lowest, which indicates the applicability of the clustering algorithm based on deep learning for noisy diffraction patterns.

Based on these results for resolution and variance, the clustering algorithm based on deep learning has better performance when dealing with low signal-to-noise ratio diffraction patterns. Therefore, it is reasonable and credible to analyse the radiation damage of mitochondria through this method.

## 3. Results and discussion

### 3.1. Analysis of radiation-induced changes in diffraction patterns

**Figure 5:**
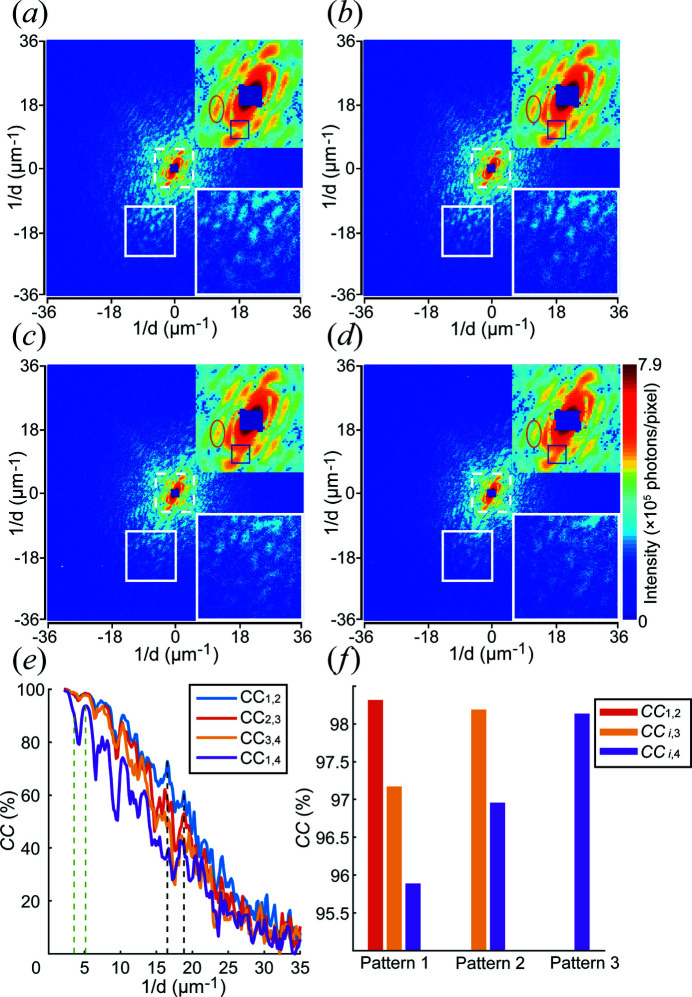
(*a*)–(*d*) X-ray diffraction patterns of the mitochondrion at different cumulative radiation doses after 3×3 binning and deconvolution. The images in the upper right corner are magnifications of the dashed squares in the centres of the patterns and the images in the lower right corner are magnifications of the solid squares in the patterns. The red ellipses and blue squares correspond to the region bounded by the two green dashed lines in panel (*e*) with a frequency range from 3.5 to 5.2 µm^−1^. The white squares correspond to the area between the two black dashed lines in panel (*e*) and the frequency in this range is from 16.5 to 18.9 µm^−1^. (*e*) The cross correlation (CC) plotted as a function of the resolution shell between two given patterns. (*f*) The CC between the three patterns. The abscissa represents the CC between the corresponding pattern and others. For example, the yellow bar in the group labelled ‘Pattern 2’ represents the CC between the second and third diffraction patterns.

Fig. 5 ▸(*a*) shows the diffraction pattern of the mitochondrion recorded with the lowest cumulative radiation dose. The diffraction speckles are well defined, whether in the low-scattering-angle region of the centre or the high-scattering-angle region in the periphery. As the radiation dose increases, some speckles lose contrast. Since the patterns were obtained over accumulating dose, the structure of the sample probably changed over the course of each exposure. Continuous changes in the electron-density distribution during the exposure reduce the contrast in a similar way to illumination by partially coherent radiation (Quiney & Nugent, 2011 ▸).

The red ellipses in the magnified area in the upper right corner of each pattern highlight a speckle where the intensity gradually decreases with increasing dose, indicating that some internal structures of the mitochondrion have gradually disappeared. On the other hand, the blue square shows a region between speckles where the intensity increases with increasing dose, making the two adjacent speckles connect. Both the red ellipse and blue square are in low-frequency areas, and the change in speckles is caused by modifications of the coarse internal structures of the mitochondrion, which should in principle be observable.

The white squares in the lower right corners of the patterns in Fig. 5 ▸ show a magnified view of the highlighted regions at somewhat higher frequencies than discussed above. It can be seen that some diffraction speckles gradually disappear from these regions as the dose increases. In Figs. 5 ▸(*a*) and 5 ▸(*b*), the speckles are still relatively abundant and clear but have reduced significantly in Fig. 5 ▸(*c*). Compared with Fig. 5 ▸(*a*), the signal of the white square in Fig. 5 ▸(*d*) has almost completely disappeared, leaving only the signal in the upper right corner. Comparing the measurements made for cumulative doses of 30.1 and 113 MGy, the contrast of this region was found to be reduced by 26.4%.

Fig. 5 ▸(*e*) illustrates the cross correlation (CC) as a function of resolution shell, and the value between all patterns decreases as the frequency *f* increases, which indicates that the overall size and shape of the sample does not change during radiation damage, while the detailed structure does change significantly. Moreover, since the noise in the patterns is dominated by Poisson statistics, the higher the frequency, the lower the intensity and hence the worse the signal-to-noise ratio, which will also reduce the CC. The change in CC between two consecutive patterns is relatively small, especially in the low-frequency region, indicating that the morphology and overall structures of the mitochondria exhibit slow variation. For the dark magenta curve, the difference between the first and fourth patterns is dramatic, indicating that the mitochondria have changed considerably compared with the beginning. The red ellipse and blue square where the intensity changes significantly in Figs. 5 ▸(*a*) to 5(*d*) correspond to the area between the two green dashed lines in Fig. 5 ▸(*e*), where there is a drop of ∼15% in value. The frequency in this range is from 3.5 to 5.2 µm^−1^, which reflects the variation in the sample at a scale of approximately 240 nm. Approximately 19% of the intensity drop occurs in the frequency range of 16.5 to 18.9 µm^−1^, which corresponds to the white squares in Figs. 5 ▸(*a*) to 5(*d*) and a scale of approximately 56 nm.

Fig. 5 ▸(*f*) illustrates the CC of different patterns. The correlation between the first and second patterns is the highest, but the subsequent patterns also decrease during X-ray irradiation according to CC_(1, 2)_, CC_(2, 3)_ and CC_(3, 4)_, showing that the sample initially resisted radiation damage. However, as the cumulative radiation dose increases, the impact of radiation becomes increasingly serious, and the same incremental dose is seen to cause more damage. Comparing the first pattern with the others, the correlation gradually decreases with increasing radiation dose, indicating that the radiation damage is persistent.

### 3.2. Analysis of the impact of radiation on the reconstructions

**Figure 6:**
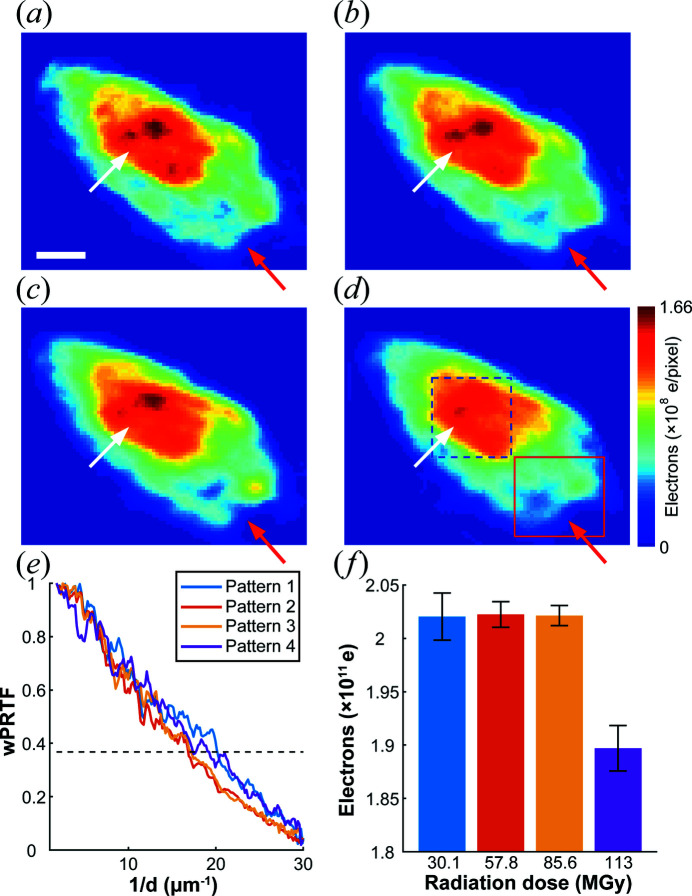
(*a*)–(*d*) Reconstructions corresponding to the first to the fourth diffraction patterns (in Fig. 5 ▸), respectively. The high- and low-density areas are outlined by the dashed blue and solid red rectangles, respectively. With increasing cumulative radiation dose, the structures in both high- and low-density areas (indicated by the white and red arrows, respectively) change. The scale bar is 200 nm. (*e*) wPRTF curves of the four diffraction patterns. The value of the dotted line is 1/e, which serves as the criterion for the resolution. (*f*) The total number of electrons calculated in the mitochondrion under different radiation doses.

The images of the mitochondrion exposed to successive incremental X-ray doses (that is, increasing cumulative doses) were reconstructed from the above four diffraction patterns and are depicted in Figs. 6 ▸(*a*) to 6(*d*). During exposure, the overall shape of the sample did not change substantially, but the internal structure changed gradually with increasing cumulative dose. For the high-density areas indicated by the white arrows in Fig. 6 ▸, the electron density continues to decrease as the radiation increased. For the low-density areas indicated by the red arrows, from the first to the third reconstruction there is no obvious loss of density. From the third reconstruction to the fourth, the structural change is the most obvious. This is consistent with the observation of an avalanche of damage at a particular critical dose (Wang *et al.*, 2009 ▸).

Based on the criterion of 1/e, the image resolutions calculated by wPRTF from the first to the fourth reconstruction were ∼49.7, 59.7, 58.5 and 57.9 nm, respectively, as shown in Fig. 6 ▸(*e*). The total number of electrons inside the mitochondrion was calculated by integrating the real-space image. Fig. 6 ▸(*f*) illustrates the total number of electrons at different radiation doses. In the first three exposures, the number of electrons was nearly constant. After the fourth exposure, approximately 6.6% of the electrons were lost.

**Figure 7:**
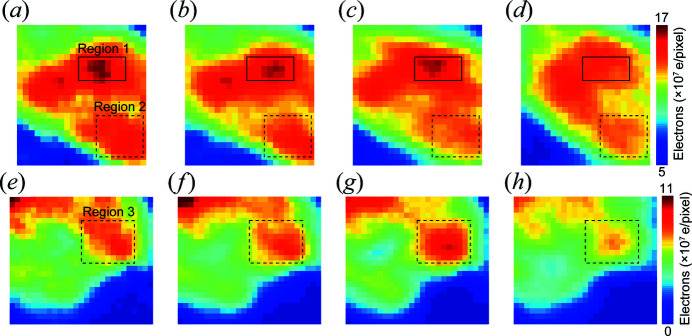
(*a*)–(*d*) Magnified views of the high-density areas of the four reconstructed images of the mitochondrion in Fig. 6 ▸, where the solid and dashed rectangles represent Region 1 and Region 2, respectively. (*e*)–(*h*) Magnified views of the low-density areas in Fig. 6 ▸, where the dashed rectangles represent Region 3.

To obtain a better understanding of the structural evolution caused by radiation, Fig. 7 ▸ shows magnified views of the mitochondrial high- and low-density areas indicated by the dashed blue and solid red rectangles in Fig. 6 ▸(*d*). Regions 1 and 2 (solid and dashed rectangles, respectively) in Figs. 7 ▸(*a*) to 7(*d*) are typical high-density areas where the electron density decreased monotonically with cumulative dose. However, the electron density in Region 3 (dashed rectangles) of Figs. 7 ▸(*e*) to 7(*f*), which represents the low-density area, increased locally during the second irradiation, which is consistent with the hypothesis of oligomerization of bio-macromolecules induced by X-ray radiation (Gianoncelli *et al.*, 2015 ▸).

**Figure 8:**
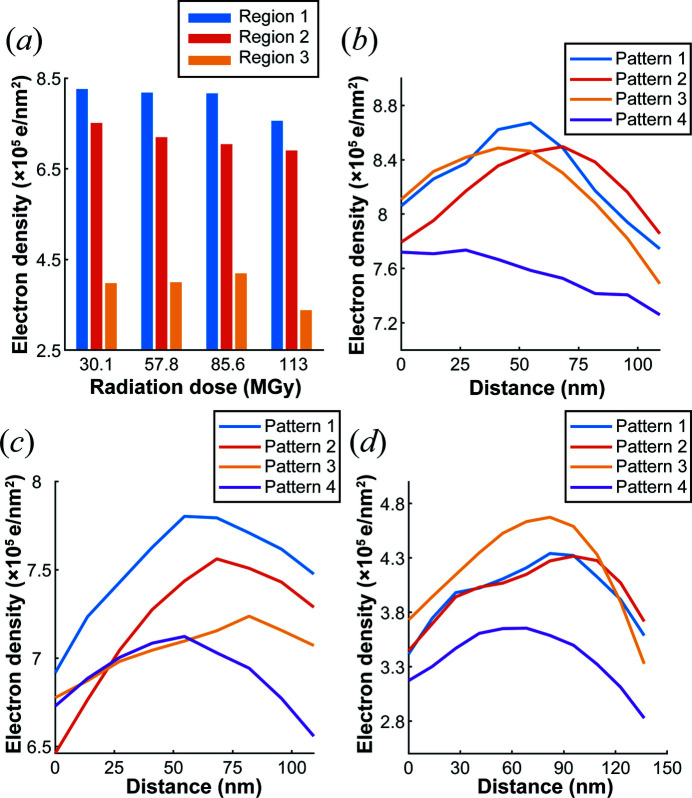
(*a*) The electron density of the regions in Fig. 7 ▸. (*b*)–(*d*) The lateral electron density calculated by vertically averaging the electron densities of Regions 1 to 3 under different doses.

Fig. 8 ▸ further quantitatively characterizes the regions in Fig. 7 ▸. Fig. 8 ▸(*a*) shows the changes in electron density in the three regions. The electron density in the high-density region decreased, while that in the low-density region first increased and then decreased. For Region 1 represented by Fig. 8 ▸(*b*), the drop was less during the first three irradiations, only approximately 2%, but the drop was approximately 10% at 113 MGy. Region 2 shown in Fig. 8 ▸(*c*) is also high density, and the density was reduced by approximately 4% each time during the first three irradiations, but after the fourth irradiation the density decreased in only part of the region. This shows that although the electron density in the high-density region always decreases overall, the variation in each part of the sample is still different. Fig. 8 ▸(*d*) clearly shows the trend that the density of the low-density region first rises and then falls. The density peaks at the third irradiation and then drops rapidly at the fourth irradiation. Also, a threshold seems to exist for mitochondria between the third and fourth exposures, corresponding to a certain dose above which a stable structure cannot be maintained and a large number of electrons are lost.

## 4. Conclusions

We have proposed a clustering algorithm based on deep learning for reconstructing images from coherent diffraction patterns of weakly scattering samples, for which it is usually difficult to obtain reliable coherent diffraction images. In comparison with other algorithms, this clustering algorithm has an advantage in dealing with noisy diffraction patterns and improving the resolution of reconstructed images.

To investigate the impact of X-ray radiation on soft biomaterials, we performed plane-wave CDI experiments on mitochondria at room temperature using synchrotron radiation. The radiation-induced changes in the diffraction patterns of the mitochondria were identified and analysed at different exposure times. Based on the reconstructions with the proposed clustering algorithm, a quantitative analysis of the electron density and its distribution in the sample showed that fine internal structure changes continuously as the radiation dose accumulates and is dramatically damaged at approximately 100 MGy, which results in diffraction patterns with reduced contrast and reduced intensity.

To protect samples and maintain their structure during X-ray irradiation, cryogenic technologies can be used to mitigate radiation damage (Huang *et al.*, 2009 ▸; Lima *et al.*, 2009 ▸; Rodriguez *et al.*, 2015 ▸). By suppressing secondary damage, these methods can improve the radiation resistance of the sample by two orders of magnitude (Beetz & Jacobsen, 2003 ▸). However, chemical bond breaking mainly caused by primary damage still exists, and occurs before the diffraction pattern changes (Coughlan *et al.*, 2017 ▸). A more reliable solution may be to use X-ray free-electron lasers (XFELs) as coherent sources, which can overcome the problem associated with radiation damage by the ‘diffraction-before-destruction’ method (Neutze *et al.*, 2000 ▸; Chapman, Barty, Bogan *et al.*, 2006 ▸). The clustering algorithm we have proposed in this work may provide a promising approach for reconstruction analysis to improve the quality of images obtained in XFEL-based plane-wave CDI experiments.

## 5. Related literature

For further literature related to the supporting information, see Arthur & Vassilvitski (2007 ▸), Chollet (2015 ▸), Cohn & Holm (2020 ▸), Culjak *et al.* (2012 ▸), Fienup (1982 ▸), Hattanda *et al.* (2014 ▸), He *et al.* (2016 ▸), Jolliffe & Cadima (2016 ▸), Lloyd (1982 ▸), Pedregosa *et al.* (2011 ▸), Rother *et al.* (2004 ▸), Russakovsky *et al.* (2015 ▸) and Simonyan & Zisserman (2014 ▸).

## Supplementary Material

Additional information on reconstruction and dose estimation. DOI: 10.1107/S2052252521012963/cw5032sup1.pdf
