# Supplementary material for: Quantitative analysis of the effect of radiation on mitochondria structure using coherent diffraction imaging with a clustering algorithm
Source: IUCrJ. 2022 Jan 21;9(Pt 2):223–30. doi: 10.1107/S2052252521012963 (PMC8895015; doi:10.1107/S2052252521012963)
Supplement: Supplementary file 1 [file m-09-00223-sup1.pdf]

IUCrJ

Volume 9 (2022)

Supporting information for article:

Quantitative analysis of the effect of radiation on mitochondria structure using coherent diffraction imaging with a clustering algorithm

Dan Pan, Jiadong Fan, Zhenzhen Nie, Zhibin Sun, Jianhua Zhang, Yajun Tong, Bo He, Changyong Song, Yoshiki Kohmura, Makina Yabashi, Tetsuya Ishikawa, Yuequan Shen and Huaidong Jiang

S1. Phase retrieval and image reconstruction

The reconstruction process for the first diffraction pattern was shown in detail as an example, because the parameters and processes used in the other three patterns were the same. After setting α = (160, 140, 120, 100, 85, 70, 55, 40, 30, 20) and β = 0.9 (α and β are the parameters of Gaussian kernels and real-space limit), a total of m = 1,000 different initial phase reconstructions with 69×83 rectangular support were performed to generate a tight support. During the iterations, the error between the calculation and experiment was monitored by an R factor (R_f) (Sekiguchi et al., 2016),

$$R_f = \frac{\sum_{\mathrm{u,v}}\left||F_c(u,v)| - |F_e(u,v)|\right|}{\sum_{\mathrm{u,v}}|F_e(u,v)|}, \tag{S1}$$

where u and v are frequencies, $|F_c(u,v)|$ and $|F_e(u,v)|$ are the Fourier modulus of the calculation and experiment, respectively. The OSS algorithm contained a total of 2000 iterations, and every 200 iterations was a step. Each step updated α to make the Gaussian kernel sharper for smoother filtering, and the reconstruction with the smallest R_f was selected as the input for the next step. However, for the high-noise diffraction patterns in this paper, the changes in α in the first few steps were sharply. If these steps also took the reconstructions with the smallest R_f as the input, the reconstruction performance was poor, so the last reconstructions in the first i steps were directly used as the input for the next step; here i = 3. In each of the remaining steps, a GrabCut was used to generate new support representing the approximate outline of the object (Rother et al., 2004). GrabCut is a commonly used image segmentation algorithm that needs nothing in addition to setting a rectangular area in advance. Outside this rectangular area is completely background and no object. Based on the characteristics of the external background, the algorithm separates the target and background in the area. Fortunately, the rectangular support set in the initial iteration could be used as the GrabCut input. After 10 steps, another one step of error reduction (ER) was implemented to further prevent stagnation problems (Fienup, 1982). In the end, rough electron density maps were obtained. By averaging the tens of reconstructions (here we averaged 24 reconstructions) with the smallest R_f and using GrabCut, a tight support could be generated. In order to prevent the support from being too compact and cut to the sample, the tight support was dilated by 3 pixels.

After the first generation of reconstructions mentioned above, the next generation would start with the newly generated tight support to improve the quality of imaging, while remain other parameters unchanged. The results of the second generation would be the final reconstructions and be used for clustering.

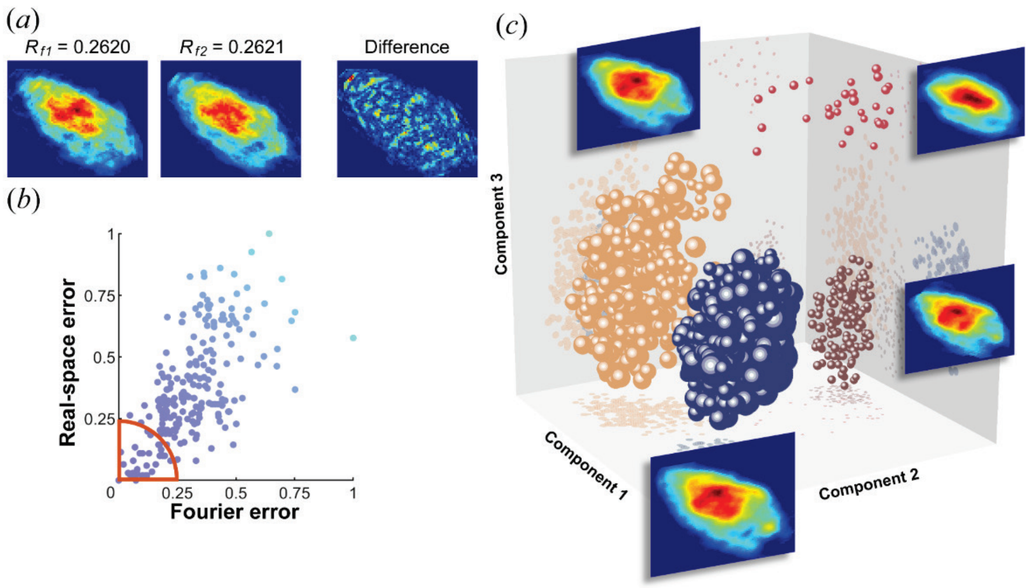

Figure S1 (*a*) Two independent reconstructions from the same diffraction pattern with similar R_f values and their variance. (*b*) Scatter plot for the cluster with the smallest error. The 24 reliable solutions nearest to the origin in the red fan-shaped area were selected, and the averaged image was used as the final imaging result. (*c*) Clustering of one thousand reconstructions from the third pattern (Fig. 1*b*) using VGG16. Reconstructions were clustered into 4 clusters, each ball represents a reconstruction, and its volume is related to R_f. The smaller the error, the larger the volume.

Normally, after hundreds of iterations, dozens of reconstructions with the smallest R_f were averaged as the final reconstructed image. However, for soft biological samples, especially for organelles without supporting structures, a weak signal leads to a poor signal-to-noise ratio in the diffraction patterns, as shown in Fig. 1(*d*). This, together with the missing data at the centre of the diffraction patterns, makes the reconstructed structures different even if the R_f is similar, as shown in Fig. S1(*a*). When these different structures are involved in averaging, high-density regions are suppressed and low-density regions are enhanced, resulting in distortion of the electron density of the final imaging result.

At present, a few clustering methods have been tested in the field of CDI. For instance, Hattanda *et al.* introduce k-means in the iterations to revise the real-space support constantly (Hattanda *et al.*, 2014). Van der Schot and co-authors design a method based on errors of real and reciprocal space (van der Schot *et al.*, 2015). Sekiguchi *et al.* propose a clustering method named ASURA (Sekiguchi *et al.*, 2016). They perform PCA and k-means both in support estimation and phase retrieval for fine structures. In this study, we investigated ConvRe, a clustering algorithm based on deep learning, and the flow chart is shown in orange in Fig. 2. Through the whole process, the extraction of features is more important, which will affect the final clustering performance.

First, all of the reconstructions were scaled up from 83×69 pixels to 224×224 pixels (through **cv2.resize**) (Culjak *et al.*, 2012) since the weights of convolutional neural networks (CNNs) used were pre-trained on the ImageNet database and only able to process images with the same size as training (Russakovsky *et al.*, 2015). The reason for choosing ImageNet is that its database contains millions of images, and the weights obtained by training with such a large database are highly reliable. Then, by the **keras.applications** module (Chollet, 2015), the VGG16, VGG19, and ResNet50 networks were used for feature extraction to find the best performing network for these reconstructions (Simonyan & Zisserman, 2014, He *et al.*, 2016). The fully connected layers were removed and the outputs were computed from a series of convolution layers. After that, there were 25088 (for VGG16 and VGG19) or 100352 (for ResNet50) features in each reconstruction, which was too large for clustering; therefore, principal component analysis (PCA) was performed through **sklearn.decomposition.PCA** (Pedregosa *et al.*, 2011) to reduce the dimensions (Jolliffe & Cadima, 2016). Ninety-nine percent of the variance in the data were retained, ensuring that the information was not lost while reducing the dimensionality. This step reduced the features to several hundred components and improved the regularity among features. The features after PCA are called the principal components, and the principal components at the top of the list contain more sample information. For example, there were 430 principal components after PCA for ResNet50, and the first three principal components contained 29.8% of the features. Therefore, by making a scatter plot of the first three principal components, the number of clusters k that needs to be set next could be determined.

Finally, the reconstructions were clustered into k clusters by k-means (through **sklearn.cluster.KMeans**), which is one of the most popular clustering algorithms due to its simplicity and efficiency (Lloyd, 1982). For better performance and efficiency, we performed k-means++, which keeps the initial k centroids away from each other (Arthur & Vassilvitskii, 2007). Cohn and Holm indicate the accuracy of the above process through VGG16 as the feature extraction network (Cohn & Holm, 2020). Although the network depth of VGG16, VGG19, ResNet50 is gradually deepening and the theoretical performance is becoming stronger, it is still necessary to compare to choose the most suitable network.

Table S1 The clustering results of Pattern1. The left side in the brackets represents the number of reconstructions included in the cluster, and the right side represents the average R_f of the reconstruction.

	**Cluster 1**	**Cluster 2**	**Cluster 3**	**Cluster 4**
**VGG16**	(70,0.2663)	(231,0.2632)	**(473,0.2620)**	(226,0.2628)
**VGG19**	(195,0.2631)	**(311,0.2620)**	(252,0.2632)	(242,0.2632)
**ResNet50**	(273,0.2631)	(247,0.2633)	**(239,0.2621)**	(241,0.2629)

Table S1 shows the results of three different networks after clustering. The cluster with the smallest R_f of each network is in bold. Among them, the results obtained by VGG16 had the largest number of reconstructions while having the smallest error, which was the most suitable network for Pattern1. Here, the number of reconstructions included in the cluster was also used as a criterion for selecting the cluster. This is because under normal circumstances, the correct results tend to appear the most frequently, and this criterion is also used in XFEL data analysis (van der Schot *et al.*, 2015, Sekiguchi *et al.*, 2016).

However, if the reconstructions in this cluster were selected indiscriminately and averaged as the imaging result, both the resolution and the contrast would be poor. Therefore, the cross correlation (CC) was introduced,

$$\mathrm{CC}_{(i,j)}=\frac{\sum_{(x,y)}\left(O_i(x,y)-\overline{O_i}\right)\left(O_j(x,y)-\overline{O_j}\right)}{\sqrt{\sum_{(x,y)}\left(O_i(x,y)-\overline{O_i}\right)^2}\sqrt{\sum_{(x,y)}\left(O_j(x,y)-\overline{O_j}\right)^2}}, \tag{S2}$$

where $O_i(x,y)$ and $\overline{O_i}$ are the ith reconstruction result and its average, respectively, and $O_j(x,y)$ is the average of all reconstructions in this cluster as the comparison standard. As shown in Fig. 3(*b*), the Fourier error is the normalized R_f, where Fourier error $error_F = \left(R_f - R_{f_{min}}\right)/\left(R_{f_{max}} - R_{f_{min}}\right)$. The real-space error, $error_R = 1 - CC_{(i,j)}$, represents the disagreement between reconstructions. By taking them as the coordinates of each reconstruction, the 24 reconstructions nearest to the origin were averaged as the final imaging result, which is shown in Fig. S2. The clustering result of 4 patterns is shown in Fig. S3.

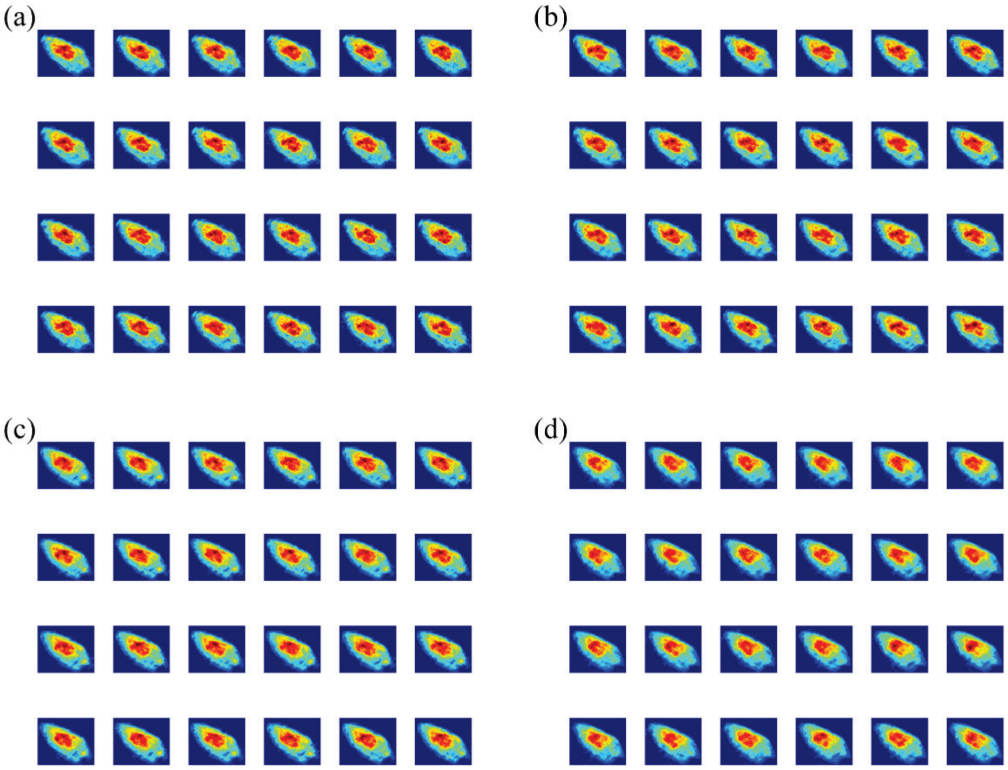

Figure S2 (*a*) ~ (*d*) The reconstructions extracted from the best clusters at radiation doses of (*a*) 30.1 MGy, (*b*) 57.8 MGy, (*c*) 85.6 MGy, and (*d*) 113 MGy.

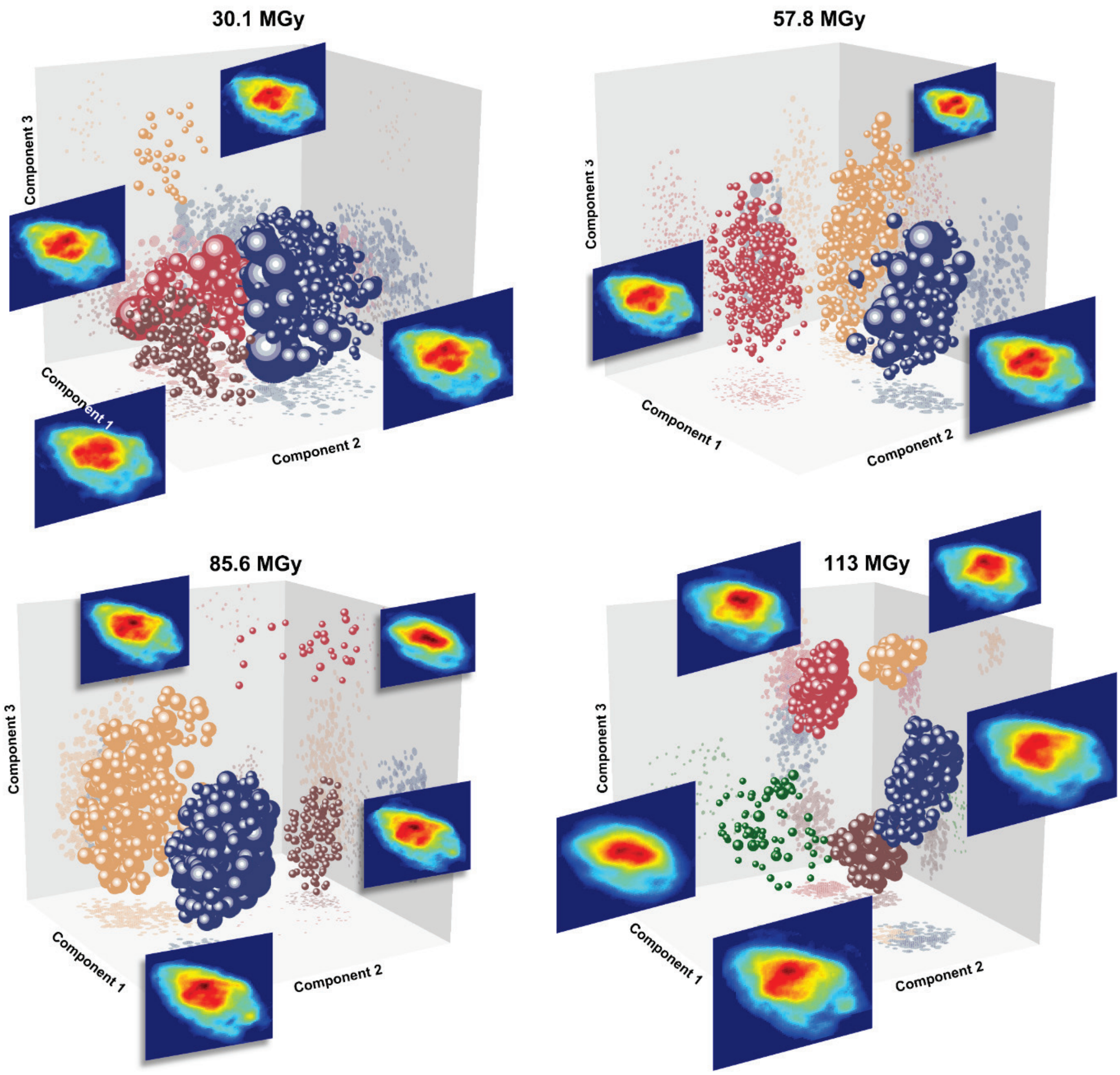

Figure S3 Clustering of one thousand reconstructions from the patterns at different radiation doses using VGG16.

S2. Radiation dose estimation for mitochondrion

To quantify the radiation dose, the incident X-ray flux has to be calculated first:

$$F_L = F_H = \frac{n_e g w}{E} = 2.99\times10^9 \text{ photons/s}, \qquad (S3)$$

where F_L and F_H represent flux of LROI and HROI respectively, $n_e = 4.5\times10^5$ the number of electron counts, $g = 10^7$ the amplifier gain, and $w = 3.65$eV the energy required to create an electron-hole pair.

Then the total incident photons per projection P_T could be got. The exposure time for the LROI pattern was ~ 0.08 s/exposure with 1000 exposures and for the HROI pattern was ~ 12 s/exposure with 80 exposures.

$$P_T = F_L \times \Delta t_L + F_H \times \Delta t_H = 3.11\times10^{12} \text{ photons} \quad (S4)$$

The total incident X-ray photons per projection and unit area (through a pinhole with a diameter of 10 μm) was

$$I_0 = \frac{\eta P_T}{A} = 1.47\times10^{22} \text{ photons/m}^2 \quad (S5)$$

where η = 83.8% is the fraction coefficient and $A = 1.77\times10^{-10}$ m^2 the area of the pinhole.

Finally, the radiation dose of the first patterns could be determined by

$$Dose = I_0 \times E \times \frac{\mu}{\rho} = 3.01\times10^{7} \text{ Gy} \quad (S6)$$

where $\frac{\mu}{\rho}$ = 23.20 cm^2/g is the mass absorption coefficient of mitochondria at 5.5keV. Since only the HROI was collected for the following three patterns, the exposure time was 960s each time. In the same way, the remaining radiation dose could be calculated, which were 57.8, 85.6, and 113 MGy respectively.

Table S2 The exposure dose to the mitochondria

	Pattern1	Pattern2	Pattern3	Pattern4
**Increased dose for each exposure (MGy)**	30.1	27.7	27.8	27.4
**Total dose at the end of corresponding exposure (MGy)**	30.1	57.8	85.6	113

S3. The PRTF and wPRTF curves of diffraction patterns at different doses

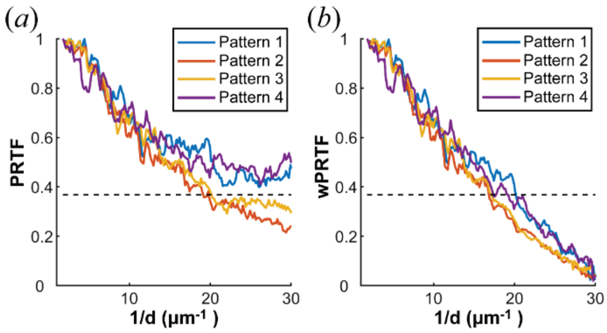

Figure S4 (a) PRTF and (b) wPRTF curves of the reconstructions obtained by OSS+ConvRe.

As shown in Fig. S4(*a*), the PRTF curves of Patterns1 and 4 show abnormal rise in the high-frequency region, which is contrary to the theory. In the high-frequency region, the signals were weak and the signal-to-noise ratio was poor. Thus, there should not be a tendency to improve the quality of reconstruction. By introducing a Wiener filter, the noise of the experimental pattern could be suppressed, thus the outliers in the PRTF were removed. The curves of Wiener-filtered phase retrieval transfer function (wPRTF) are shown in Fig. S4(*b*). The curves in the high-frequency region show a downward trend which is consistent with the theory.

S4. The variance of different methods at radiation doses of 30.1 MGy and 113 MGy

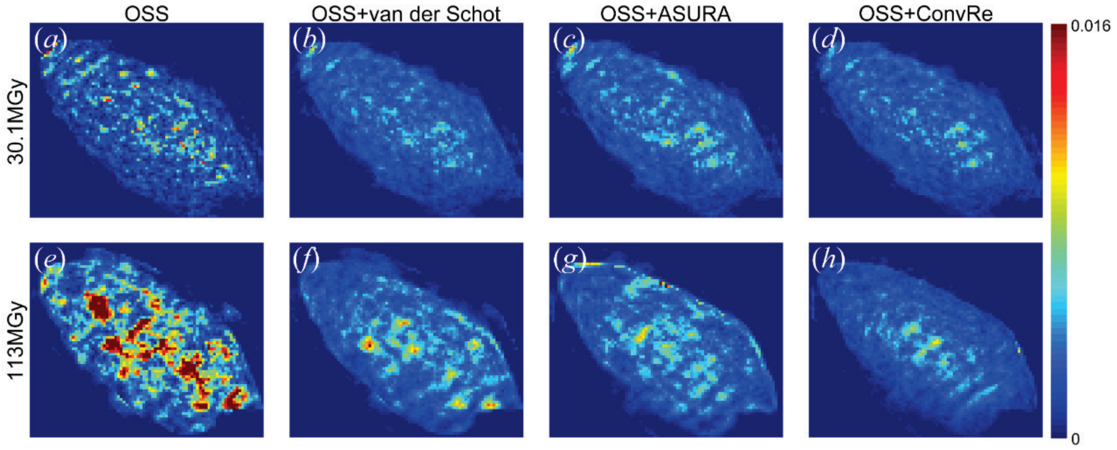

Figure S5 (*a*)~(*d*) The variance of different methods at the dose of 30.1 MGy. (*e*)~(*h*) The variance of different methods at the dose of 113 MGy.

References

Arthur, D. & Vassilvitskii, S. (2007). K-means++: The advantages of careful seeding, Proceedings of the Eighteenth Annual ACM-SIAM Symposium on Discrete Algorithms, pp. 1027–1035

Chollet, F. (2015). Keras.

Cohn, R. & Holm, E. (2020). *arXiv e-prints* arXiv:2007.08361

Culjak, I., Abram, D., Pribanic, T., Dzapo, H. & Cifrek, M. (2012). *2012 Proceedings of the 35th International Convention MIPRO*, pp. 1725-1730.

Fienup, J. R. (1982). *Appl. Opt.* **21**, 2758-2769.

Hattanda, S., Shioya, H., Maehara, Y. & Gohara, K. (2014). *J. Opt. Soc. Am. A* **31**, 470-474.

He, K., Zhang, X., Ren, S. & Sun, J. (2016). *Proceedings of the IEEE Conference on Computer Vision and Pattern Recognition*, pp. 770-778.

Jolliffe, I. T. & Cadima, J. (2016). *Philosophical Transactions of the Royal Society A: Mathematical, Physical and Engineering Sciences* **374**, 20150202.

Lloyd, S. (1982). *IEEE Transactions on Information Theory* **28**, 129-137.

Pedregosa, F., Varoquaux, G., Gramfort, A., Michel, V., Thirion, B., Grisel, O., Blondel, M., Prettenhofer, P., Weiss, R. & Dubourg, V. (2011). *the Journal of machine Learning research* **12**, 2825-2830.

Rother, C., Kolmogorov, V. & Blake, A. (2004). *ACM Transactions on Graphics* **23**, 309–314.

Russakovsky, O., Deng, J., Su, H., Krause, J., Satheesh, S., Ma, S., Huang, Z., Karpathy, A., Khosla, A., Bernstein, M., Berg, A. C. & Fei-Fei, L. (2015). *International Journal of Computer Vision* **115**, 211-252

Sekiguchi, Y., Oroguchi, T. & Nakasako, M. (2016). *Journal of Synchrotron Radiation* **23**, 312-323.

Simonyan, K. & Zisserman, A. (2014). *arXiv preprint arXiv:1409.1556*

van der Schot, G., Svenda, M., Maia, F. R. N. C., Hantke, M., DePonte, D. P., Seibert, M. M., Aquila, A., Schulz, J., Kirian, R., Liang, M., Stellato, F., Iwan, B., Andreasson, J., Timneanu, N., Westphal, D., Almeida, F. N., Odic, D., Hasse, D., Carlsson, G. H., Larsson, D. S. D., Barty, A., Martin, A. V., Schorb, S., Bostedt, C., Bozek, J. D., Rolles, D., Rudenko, A., Epp, S., Foucar, L., Rudek, B., Hartmann, R., Kimmel, N., Holl, P., Englert, L., Duane Loh, N.-T., Chapman, H. N., Andersson, I., Hajdu, J. & Ekeberg, T. (2015). *Nature Communications* **6**, 5704.
